# Biochemical, Genotoxic and Somatic responses of *Oreochromis Niloticus* Exposed to Enalapril Maleate in Water

**DOI:** 10.1007/s00128-026-04322-y

**Published:** 2026-10-12

**Authors:** Allana Kiara Peretti da Silva, Otávio Augusto Wolff Colaço, Yorrannys Mannes, Wanessa Algarte Ramsdorf

**Affiliations:** Laboratory of Ecotoxicology -Academic Department of Chemistry and Biology, Federal Technological University of Paraná, Curitiba, PR Brasil

**Keywords:** Bioassay, Biomarkers, Emerging Contaminant, Genotoxicity, Oxidative Stress

## Abstract

Pharmaceutical residues are increasingly detected in aquatic environments; however, information regarding the ecotoxicological effects of antihypertensive drugs such as enalapril maleate remains limited, particularly in fish. This study aimed to evaluate the effects of aqueous exposure to EM on somatic, biochemical, and genotoxic biomarkers in *Oreochromis niloticus* under controlled laboratory conditions. A 21-day subchronic bioassay was conducted using fish exposed to EM-contaminated water at nominal concentrations of 100, 500, 1000, and 2000 µg/L, alongside a control group. Genotoxic effects were evaluated using the comet assay and micronucleus test, while biochemical biomarkers - including acetylcholinesterase, catalase, glutathione S-transferase, and lipid peroxidation - were assessed to investigate oxidative stress and potential neurotoxicity. Somatic condition was also analyzed as an indicator of overall organism health. The results demonstrated genotoxic effects, evidenced by DNA damage. Biochemical analyses revealed alterations consistent with oxidative stress and potential neurotoxic responses, while somatic indices showed minor but detectable health changes. Although the observed damage levels were relatively low, these findings indicate that continuous exposure to EM and its byproducts can induce sublethal physiological disturbances with potential long-term consequences for aquatic organisms. Overall, this study highlights the relevance of ecotoxicological monitoring of EM in aquatic systems. The observed biological responses underscore its potential ecological risk, and future studies should incorporate preliminary stability assessments and evaluate long-term effects and degradation products to better understand the environmental impact of this antihypertensive drug.

## Highlights

The drug enalapril maleate (EM) is considered an emerging contaminant because it is found in water bodies, but its concentrations are not monitored.

In a 21-day bioassay, fish exhibited changes in biomarkers, even at low concentrations (in the order of nanograms per liter).

The observed effects included genotoxicity, oxidative stress, and neurotoxic effects.

## Introduction

Research on emerging contaminants (ECs) has gained increasing relevance in recent decades due to their continuous release into the environment and their potential risks to ecosystems and human health (Gavrilescu et al. 2015). ECs encompass a variety of chemical substances that are not routinely monitored or fully regulated but may exert biological effects even at low concentrations (Montagner et al. 2017). Conventional wastewater treatment plants are not specifically designed to remove many of these compounds, allowing their persistence and dissemination in aquatic environments (Pescara, 2014).

Pharmaceuticals represent an important class of emerging contaminants and have been widely detected in surface waters worldwide, typically at concentrations ranging from ng/L to µg/L (Calamari et al. 2003; Wiegel et al. 2004; Fent et al. 2006; Pérez et al. 2018; Rojo et al. 2021). These substances enter aquatic systems primarily through domestic wastewater effluents following human excretion, hospital discharges, pharmaceutical manufacturing effluents, and improper disposal of unused medications (Daughton and Ternes 1999; Hughes, Kay, Brown, 2013). Their continuous input raises concerns regarding chronic exposure and sublethal effects on non-target aquatic organisms.

Among these compounds, EM is a widely prescribed antihypertensive drug belonging to the angiotensin-converting enzyme (ACE) inhibitor class. It is extensively used in the treatment of arterial hypertension and heart failure, conditions with high global prevalence. Enalapril and its active metabolite enalaprilat have been reported in different aquatic matrices worldwide, including surface waters, wastewater effluents, and hospital discharges. Studies conducted in Europe, Asia, and South America have detected these compounds at concentrations ranging from ng/L to low µg/L (Pérez et al. 2018; Rojo et al. 2021; Zhang et al. 2020; Navon et al. 2024; Jaukovic et al. 2014). Although these concentrations are generally considered environmentally low, the continuous input of antihypertensive drugs into aquatic ecosystems raises concerns regarding long-term and sublethal effects on non-target organisms, especially under chronic exposure scenarios. However, ecotoxicological data addressing the mechanistic effects of enalapril and its degradation byproducts at the biochemical and genetic levels are still scarce, particularly in tropical fish species, highlighting an important knowledge gap addressed by this study.

Given the ecological relevance of pharmaceutical contamination and the scarcity of data addressing the effects of EM and its degradation byproducts on fish species, this study aimed to evaluate the subchronic effects of EM in *O. niloticus* using biochemical, genotoxic, and somatic biomarkers.

## Methods and Materials

### Test Organisms

Juvenile *Oreochromis niloticus* were commercially obtained and used as test organisms. At the beginning of the experiment, fish presented a mean body weight of approximately 8 g and a mean total length of 5–6 cm. Prior to exposure, organisms were acclimated for 21 days in aerated tanks containing filtered and dechlorinated water under controlled laboratory conditions. During acclimation and exposure, fish were fed daily with commercial feed appropriate for the species.

All experimental procedures were approved by the Ethics Committee on the Use of Animals of the Federal University of Technology - Paraná (UTFPR), under protocol number 23064.042797/2023-78. The choice of *O. niloticus* was based on its ecological relevance, ease of handling, rapid growth, and frequent use in ecotoxicological studies.

## Bioassay

Enalapril maleate (purity 99.7%) was obtained from a certified chemical supplier (Delaware Ltda.). Stock solutions were prepared by directly dissolving EM in dechlorinated water, without the use of organic solvents. Working solutions were freshly prepared by dilution of the stock solution to achieve nominal concentrations of 100, 500, 1000, and 2000 µg/L.

The bioassay consisted of five experimental groups (control and four exposure treatments), each containing 14 individuals, and was conducted over 21 days under semi-static conditions. Throughout the exposure period, water quality parameters were monitored daily, and remained stable throughout the experiment (temperature: 22–26 °C; dissolved oxygen: 4.0–6.5 mg/L; pH: 6.7–7.4). Water was renewed daily, with reapplication of EM to maintain nominal exposure concentrations. No mortality was observed throughout the experimental period. At the end of exposure, fish were anesthetized with benzocaine (10%) and euthanized by spinal cord section. Total length and body mass were recorded for somatic condition analysis. Blood, liver, brain, muscle, and kidney samples were collected and immediately processed or stored at –80 °C until analysis, according to the requirements of each biomarker.

## Analytical Confirmation of Exposure Concentrations

To confirm exposure concentrations and assess compound stability, water samples were collected at 15 min, 3 h, 6 h, and 24 h after preparation under experimental conditions, as well as during the bioassay (days 1, 5, and 10). Analyses were performed using liquid chromatography coupled to mass spectrometry (LC-MS). Results demonstrated progressive degradation of EM in the aqueous medium. Initial concentrations ranged from 28.47 to 30.83 ppm and decreased to 12.20–8.81 ppm after 24 h. Similar declining trends were observed during the bioassay, particularly at lower nominal concentrations. These findings indicate low stability of EM in aquatic systems, likely due to photodegradation and/or adsorption to suspended particles. Due to this instability, daily water renewal with reapplication of the contaminant was adopted and maintained nominal exposure levels throughout the experiment. Therefore, exposure concentrations reported in this study correspond to nominal values.

## Biochemical Biomarkers

Each biochemical test followed a specific methodology: Keen et al. (1976) for GST, Jiang et al. (1992) for LPO, Aebi (1984) with adaptations by Vicentini et al. (2022) for CAT, and Ellman et al. (1961), adapted by Silva de Assis (1998), for AChE. The biomarkers GST, LPO, and CAT were assessed in liver tissue, while AChE activity was analyzed in brain and muscle cells. Absorbance readings were used to quantify enzymatic activity and lipid peroxidation levels.

## Genetic Biomarkers

Genotoxic effects were assessed using the comet assay (Östling and Johanson 1984; Singh et al. 1988) and the piscine micronucleus test (Heddle 1973; Schmid 1975). The comet assay was performed using blood and kidney cells, with 100 cells analyzed per slide and classified into damage classes to calculate a damage score. The micronucleus test was conducted on peripheral blood erythrocytes, with 1000 cells evaluated per individual under blind conditions.

### Somatic Biomarker

Somatic condition was evaluated using Fulton’s Condition Factor (K), calculated from individual body weight (W, g) and total length (L, cm) according to the equation K = W / L³, as described by Fulton (1902) and standardized by Ricker (1975).

## Statistical Analyses

Data were analyzed using BioEstat^®^ and GraphPad Prism^®^. Genetic biomarkers were evaluated using the nonparametric Kruskal-Wallis test followed by Dunn’s post hoc test. Biochemical and somatic data were analyzed by one-way ANOVA followed by Bonferroni’s post hoc test. Differences were considered significant at *p* < 0.05.

## Results

### Catalase (CAT)

**Fig. 1:**
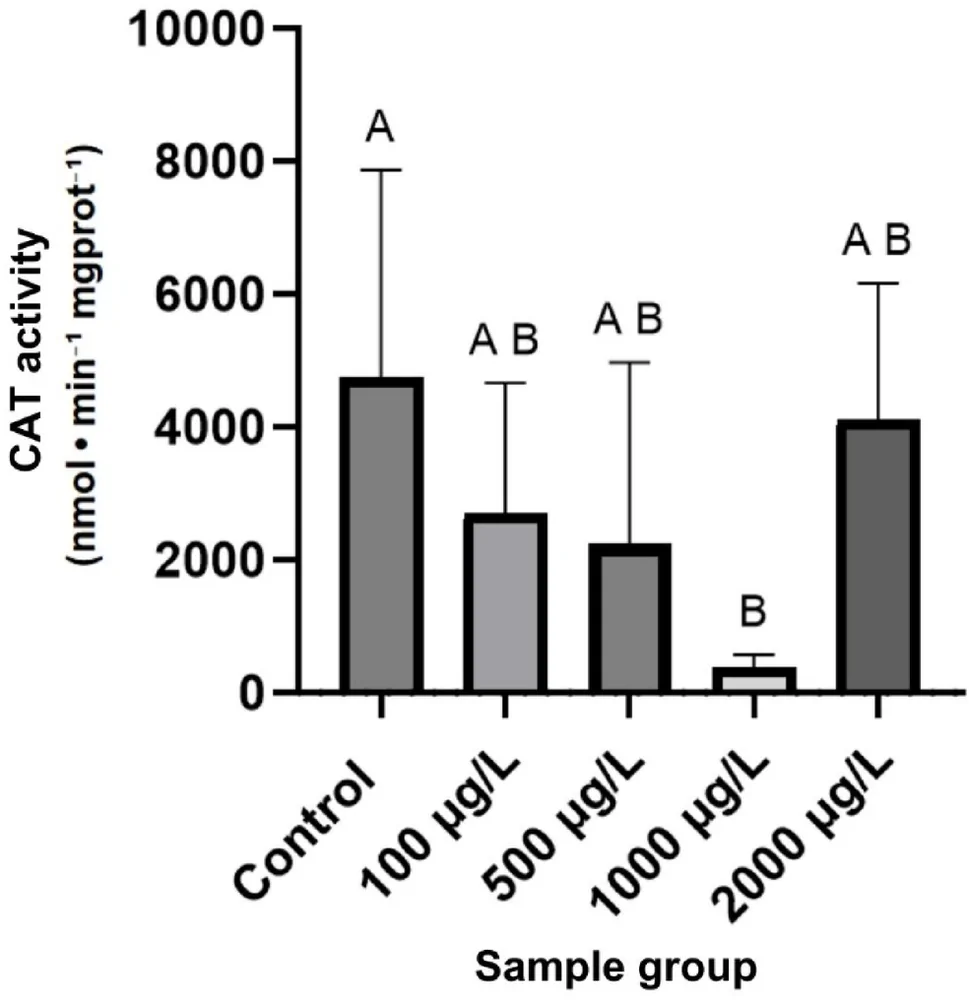
Catalase (CAT) activity in *O. niloticus*. Groups with the same letters indicate that there is no statistically significant difference between them, and different letters indicate that there is a difference. The error bars represent mean ± standard deviation (SD)

CAT activity in *O. niloticus* showed a statistically significant difference among the experimental groups (*p* = 0.0214), with a notable reduction in enzymatic activity observed in the group exposed to the 1000 µg/L concentration (Fig. 1). The control group exhibited the highest enzymatic activity among all treatments, suggesting the presence of a basal antioxidant response that was absent in the exposed groups.

### Acetylcholinesterase (AChE)

**Fig. 2:**
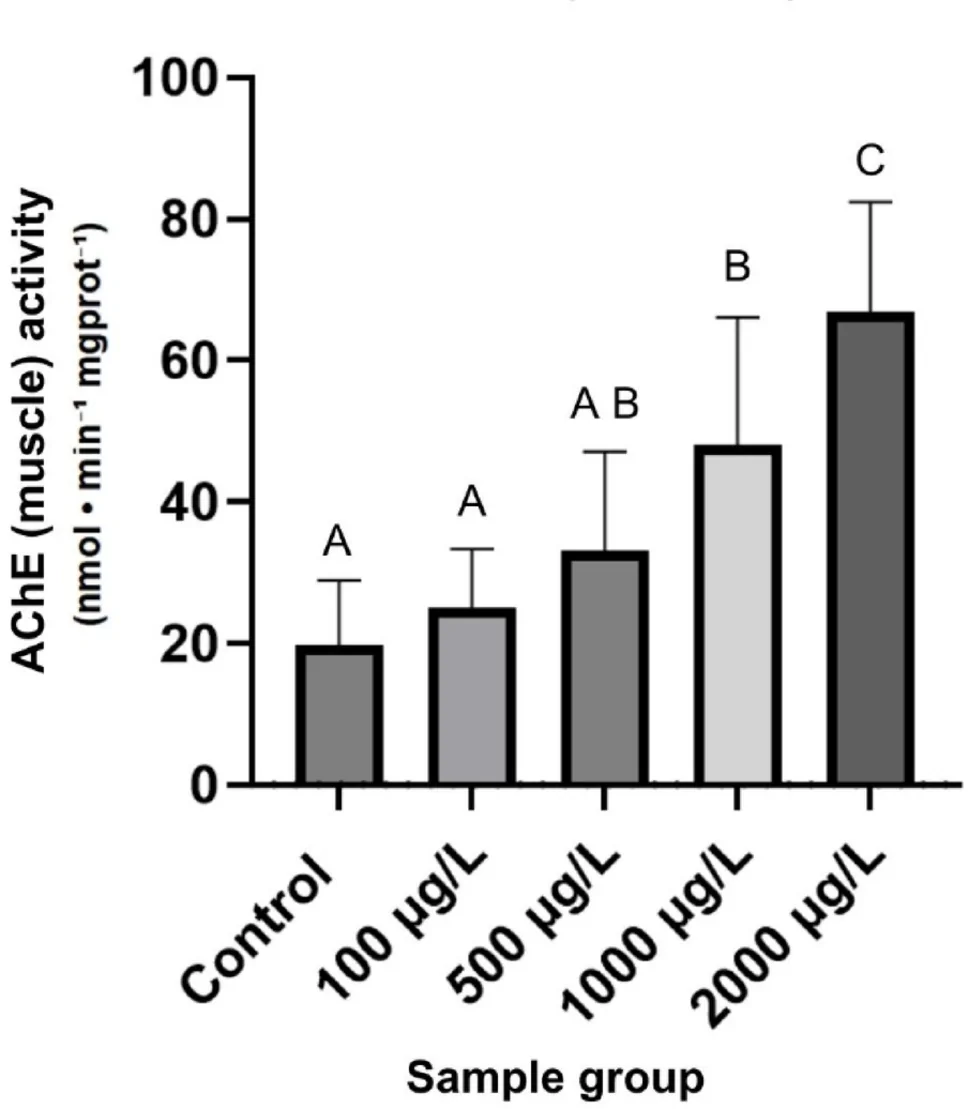
Acetylcholinesterase (AChE) activity in muscle tissue of *O. niloticus*. Groups with the same letters indicate that there is no statistically significant difference between them, and different letters indicate that there is a difference. The error bars represent mean ± standard deviation (SD)

**Fig. 3:**
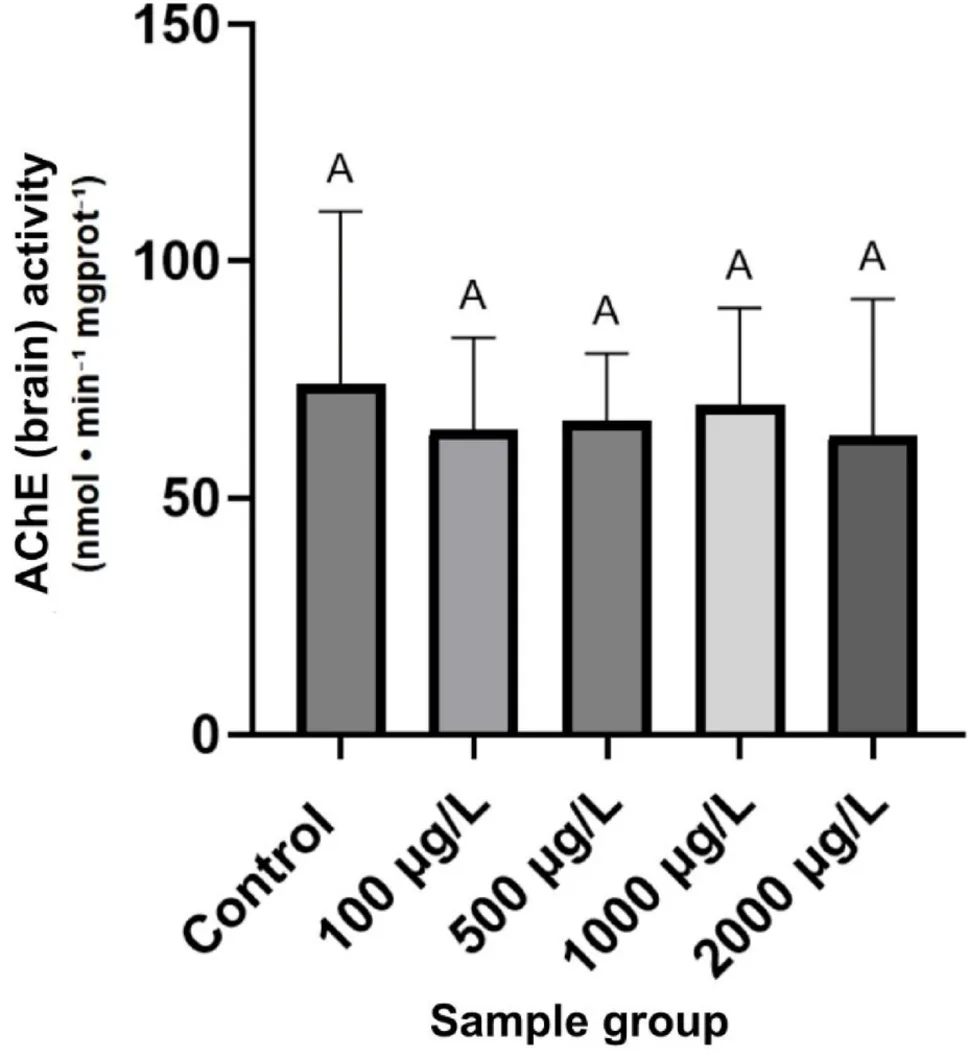
Acetylcholinesterase (AChE) activity in brain tissue of *O. niloticus*. Groups with the same letters indicate that there is no statistically significant difference between them, and different letters indicate that there is a difference. The error bars represent mean ± standard deviation (SD)

AChE activity in the muscle tissue showed a statistically significant difference among groups (*p* < 0.0001), with a concentration-dependent increase in enzymatic activity (Fig. 2). In brain tissue, no statistically significant differences in AChE activity were observed among the groups (*p* = 0.8284), with similar values across all treatments (Fig. 3).

### Lipid Peroxidation (LPO)

**Fig. 4:**
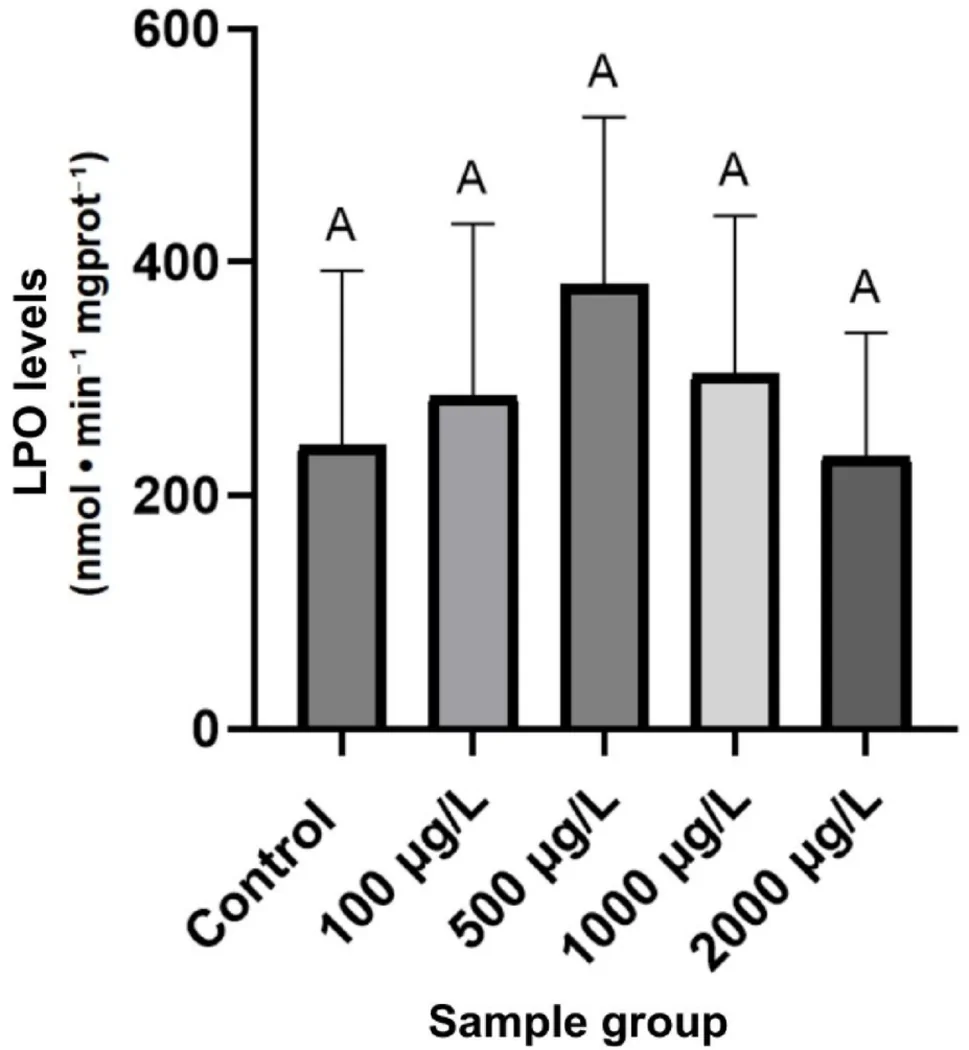
Lipoperoxidation (LPO) assessment in *O. niloticus*. Groups with the same letters indicate that there is no statistically significant difference between them, and different letters indicate that there is a difference. The error bars represent mean ± standard deviation (SD)

No statistically significant differences were observed in oxidized lipid levels among the sample groups (*p* = 0.0766) (Fig. 4). These results suggest that, under the tested conditions, EM exposure was not sufficient to induce a significant increase in lipid peroxidation in *O. niloticus.*

### Glutathione-S-Transferase (GST)

**Fig. 5:**
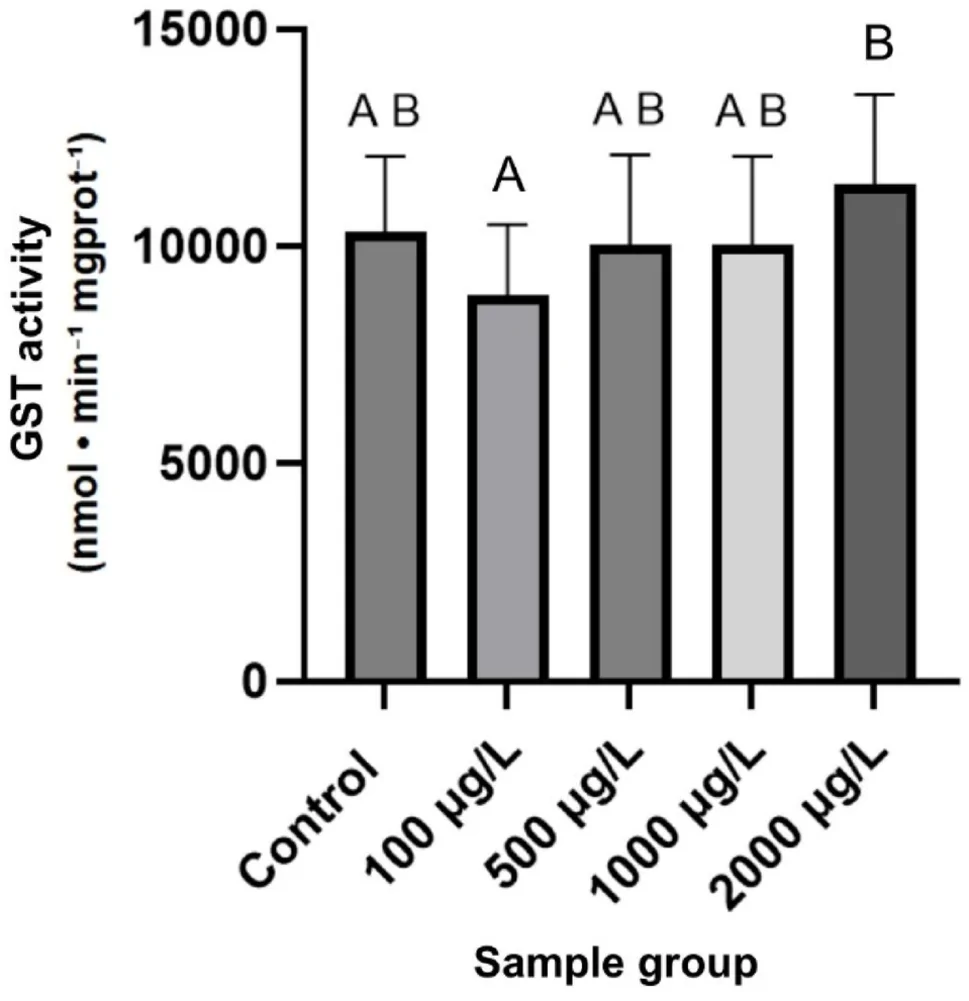
Glutathione-S-transferase (GST) activity in *O. niloticus*. Groups with the same letters indicate that there is no statistically significant difference between them, and different letters indicate that there is a difference. The error bars represent mean ± standard deviation (SD)

GST activity in *O. niloticus* showed a statistically significant difference among groups (*p* = 0.0242), with increased activity in the groups exposed to EM (Fig. 5). However, no significant difference was found between the exposed groups and the control.

### Comet Assay

**Fig. 6:**
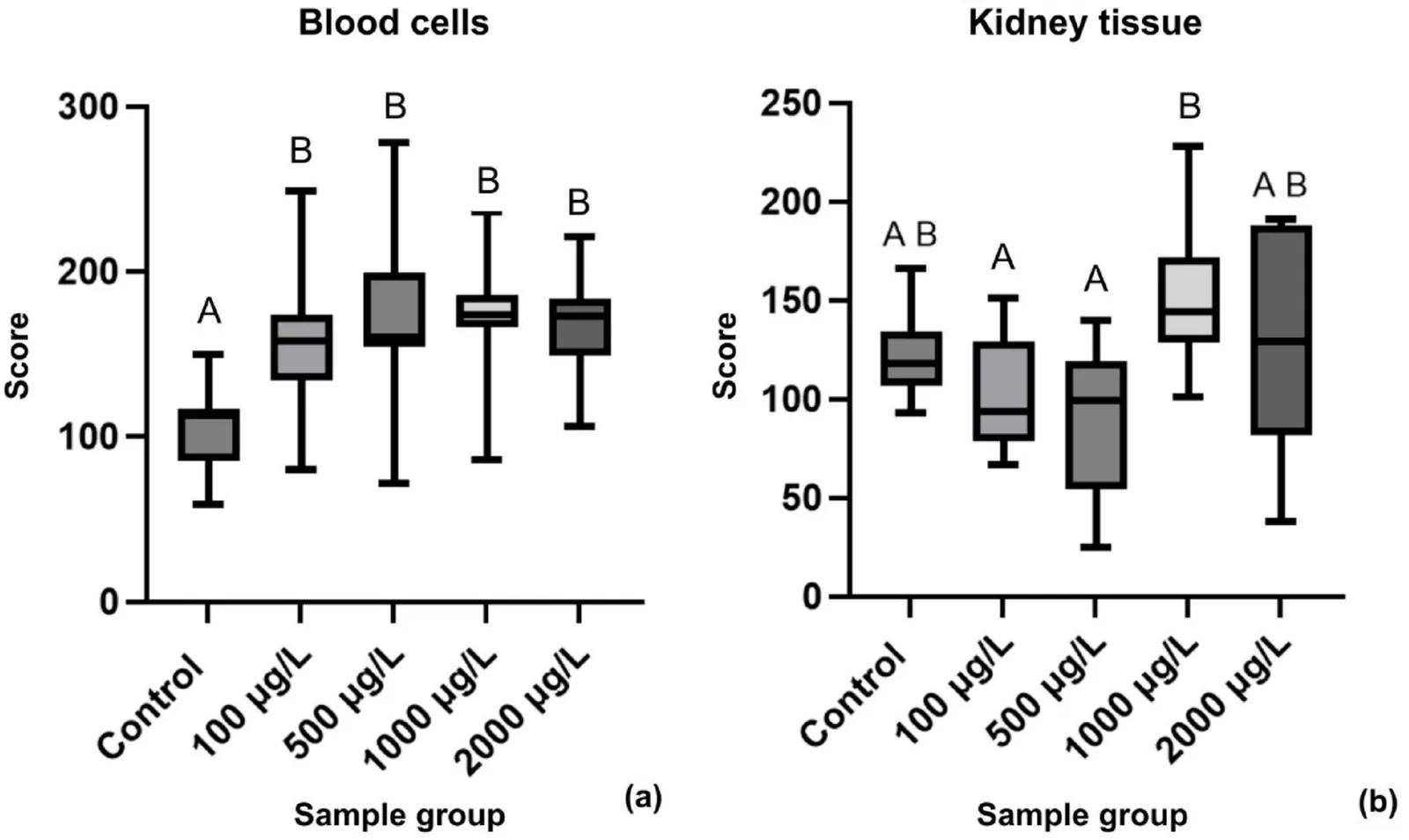
Comet assay scores found in blood cells (**a**) and kidney tissue (**b**) of *O. niloticus*, respectively. Groups with the same letters indicate that there is no statistically significant difference between them, and different letters indicate that there is a difference. The error bars represent mean ± standard deviation (SD)

The results of the comet assay showed a statistically significant difference for both analyzed tissues (*p* < 0.05). However, when compared to the negative control, only the blood cells exhibited a significant difference in damage scores, with *p* = 0.0008, while renal cells showed *p* = 0.0215 (Fig. 6a and b).

### Fish Micronucleus Test

**Fig. 7:**
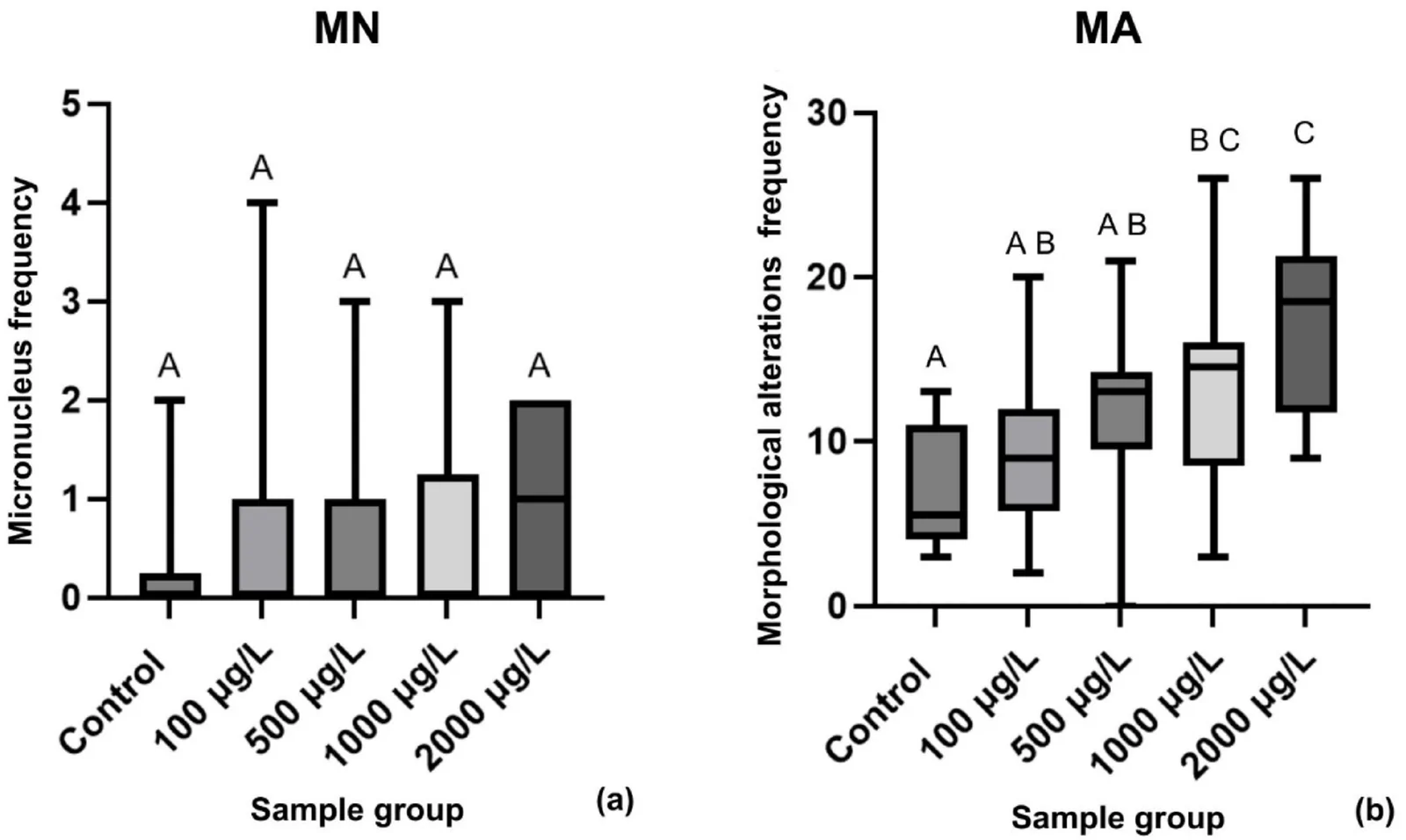
Frequencies of micronuclei (**a**) and nuclear morphological alterations (**b**) found in *O. niloticus*. Groups with the same letters indicate that there is no statistically significant difference between them, and different letters indicate that there is a difference. The error bars represent mean ± standard deviation (SD)

The analysis of the cells revealed an increase in the frequency of nuclear morphological alterations (NMA) in the groups exposed to EM, with a rising median as the contaminant concentration increased. The difference was statistically significant (*p* < 0.0001) (Fig. 7). These data indicate that EM has genotoxic potential, inducing nuclear alterations in the blood cells of *O. niloticus.* For the micronuclei (MN) found, although a trend of increase was observed in the exposed groups, the results for the exposed groups did not show a statistically significant difference compared to the control (*p* = 0.4842), suggesting a low mutagenic potential at the evaluated concentrations.

### Fulton’s Condition Factor

**Fig. 8:**
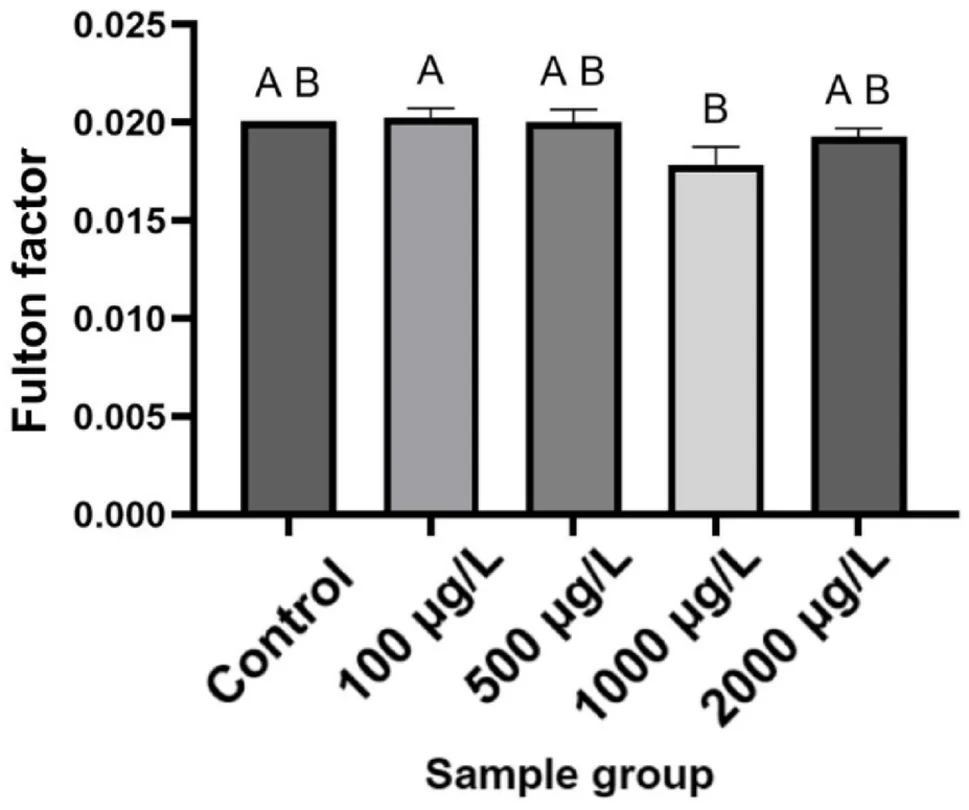
Comparison between the Fulton Factor found for each sample group after exposure. Groups with the same letters indicate that there is no statistically significant difference between them, and different letters indicate that there is a difference. The error bars represent mean ± standard deviation (SD)

Fulton’s condition factor (K) was used as an indicator of somatic condition before and after exposure. At the end of the bioassay, a significant difference among groups was observed (*p* = 0.03), with a tendency toward reduced K values at higher enalapril maleate concentrations, although no individual exposed group differed significantly from the control. A significant decrease in K was observed when comparing pre- and post-exposure values (*p* = 0.0001), indicating an overall negative effect of exposure on fish condition (Fig. 8).

## Discussion

Considering the constant introduction of pharmaceutical products, such as EM, into the environment through the discharge of effluents from industries, hospitals, and residences, our experimental methodology involved the daily renewal of EM. However, analytical determination confirmed that enalapril maleate is unstable in aqueous medium, undergoing rapid degradation. Despite daily renewal, the partial degradation suggests that the biological responses observed in this study may reflect both the effects of the original compound, added daily, and its degradation products. This exposure to EM and its degradation products is consistent with what is observed in the impacted natural environment, given that EM and its byproducts are continuously introduced into the environment.

The reduction in catalase enzymatic activity may be related to the overload of reactive oxygen species (ROS) induced by EM, surpassing the antioxidant capacity of the exposed organisms. This pattern is consistent with that described by Zagatto and Bertoletti (2008), who associate ROS accumulation with cellular damage in aquatic organisms.

Previous studies have also reported alterations in CAT activity associated with contaminant exposure. Pérez et al. (2018) observed a reduction in enzymatic activity in the brain and gills of *Prochilodus lineatus* exposed to EM, although an increase was recorded in the liver. Divergent results were reported by Filice et al. (2024), who found no significant alteration in CAT activity in *Mytilus galloprovincialis* exposed to EM, and by Ahmed et al. (2019), who observed increased enzyme activity in diabetic rats treated with enalapril. Additionally, Cortes-Diaz et al. (2017) reported an increase in CAT activity in *Cyprinus carpio* exposed to captopril, another ACE inhibitor, suggesting that drugs in this class may induce oxidative stress, further supporting the findings of the present study.

The AChE activity in the muscle tissue indicates a possible effect of the contaminant on the peripheral nervous system, triggering an initial adaptive response to maintain nerve impulse transmission. For the brain tissue, the result contrasts with the findings of Pérez et al. (2018), who reported inhibition of cerebral AChE in *P. lineatus* exposed to enalapril at concentrations ranging from 44.5 to 96.3 ng/L. Alterations in AChE activity may represent a response to cellular stress (Zagatto and Bertoletti 2008). No other studies evaluating AChE in fish exposed to EM were found; however, Osman and Ali (2004) reported similar patterns of enzymatic alteration in response to other xenobiotics. Despite the increase in muscular AChE activity, no behavioral changes or mortality were observed in the exposed organisms.

Although the present study did not demonstrate relevant alterations in oxidized lipid levels, Pérez et al. (2018) reported an increase in LPO in the liver and gills of *P. lineatus* exposed to enalapril, while a reduction was observed in the brain. These results suggest that the response may vary depending on the tissue and species analyzed. Additionally, Yuyen et al. (2006) observed a reduction in LPO levels in Wistar rats subjected to angiotensin-converting enzyme inhibition by captopril, indicating that this mechanism may, in some contexts, mitigate lipid peroxidation. Similar results were described by Cortes-Diaz et al. (2017), who reported oxidative stress and damage in the gills and kidneys of *Cyprinus carpio* exposed to captopril at concentrations of 1 µg/L, 1 mg/L, and 100 mg/L. Despite the similar mechanism of action between captopril and EM, it is important to note that they are distinct compounds.

The increase of Glutathione-S-transferase activity may indicate the activation of cellular detoxification mechanisms. These results corroborate the findings of Rojo (2021), who observed increased GST activity in the liver of fish exposed to a mixture of pharmaceuticals, including enalapril. Ahmed et al. (2019) also reported an increase in GST activity in the kidneys of diabetic rats treated with enalapril at 25 mg/L, indicating that this drug can induce enzymatic responses even in organisms from different taxonomic groups. Also, there is a study by Filice et al. (2024) which demonstrated that antihypertensive drugs, such as propranolol, alter GST activity in *Mytilus galloprovincialis*, increasing it at low concentrations (11 µg/L) and inhibiting it at higher concentrations (403 µg/L), in addition to promoting lipid peroxidation and AChE inhibition in the gills.

Therefore, the results obtained for all of the biochemical biomarkers suggest that EM, at the tested concentrations, induces alterations in the antioxidant and neurochemical systems of *O. niloticus*, although without lethal effects. The species showed tolerance to the contaminant, with greater sensitivity observed in muscle tissue, followed by the liver. The brain showed no significant alterations, indicating lower susceptibility to the compound.

The results of the comet assay in renal tissues, although a significant difference was observed in the overall test, there was no consistent damage pattern among the exposed groups, and none of the groups showed a statistically significant difference compared to the control. This suggests lower sensitivity of renal tissue to the contaminant, possibly because blood cells tend to exhibit toxic effects before they manifest in other organs. In blood cells, the median damage indices were significantly higher in the exposed groups compared to the control, indicating that EM can induce genotoxic damage at the studied concentrations. This result is consistent with Leão (2016), who also reported increased genotoxicity in blood cells with rising contaminant concentrations.

About the micronucleus test, our findings are consistent with the study by Leão (2016), which indicates that EM can cause genotoxic damage without inducing mutations. The results reinforce the data obtained from the comet assay, which also revealed an increase in genetic damage in blood cells. The results suggest that EM induces genetic damage at a basal level, but not to the extent of causing mutations. The genetic biomarkers demonstrated great sensitivity and consistency, and blood cells stood out as the most responsive tissue to the contaminant.

Fulton’s condition factor (K) indicates a negative impact on the nutritional and physiological status of the organisms. Although this parameter is not highly sensitive to contaminant exposure, its reduction supports the sublethal stress scenario indicated by the biochemical and genetic biomarkers (Almeida et al. 2019; Huang et al. 2020; Juárez-Juárez et al. 2017).

Although the bioconcentration of EM is reduced in neotropical fish (BCF = 0.007 to 309 µg/L in 96 h in *Cnesterodon decemmaculatus*, according to Rojo et al. 2023), which implies minimal accumulation in fish before degradation, our results reflected damage to the health of fish after exposure to EM and its degradation byproducts. The results obtained in this study indicate that EM and its degradation products can cause adverse effects in *Oreochromis niloticus*, demonstrating that the continuous introduction of EM into the aquatic environment causes genetic damage to blood cells and cellular responses indicative of oxidative stress and neurotoxicity.
